# HAD hydrolase function unveiled by substrate screening: enzymatic characterization of *Arabidopsis thaliana* subclass I phosphosugar phosphatase AtSgpp

**DOI:** 10.1007/s00425-012-1809-5

**Published:** 2012-11-24

**Authors:** José A. Caparrós-Martín, Iva McCarthy-Suárez, Francisco A. Culiáñez-Macià

**Affiliations:** Instituto de Biología Molecular y Celular de Plantas ‘‘Eduardo Primo Yúfera’’ (UPV-CSIC), Universidad Politécnica de Valencia, Ciudad Politécnica de la Innovación (CPI), C/Ingeniero Fausto Elio s/n, 46022 Valencia, Spain

**Keywords:** Abiotic and biotic stress, *Arabidopsis*, HAD superfamily, Hydrolases, Pi homeostasis, Sugar phosphatases

## Abstract

This work presents the isolation and the biochemical characterization of the *Arabidopsis thaliana* gene *AtSgpp*. This gene shows homology with the *Arabidopsis* low molecular weight phosphatases AtGpp1 and AtGpp2 and the yeast counterpart GPP1 and GPP2, which have a high specificity for dl-glycerol-3-phosphate. In addition, it exhibits homology with DOG1 and DOG2 that dephosphorylate 2-deoxy-d-glucose-6-phosphate. Using a comparative genomic approach, we identified the *AtSgpp* gene as a conceptual translated haloacid dehalogenase-like hydrolase HAD protein. *AtSgpp* (locus tag At2g38740), encodes a protein with a predicted Mw of 26.7 kDa and a pI of 4.6. Its sequence motifs and expected structure revealed that AtSgpp belongs to the HAD hydrolases subfamily I, with the C1-type cap domain. In the presence of Mg^2+^ ions, the enzyme has a phosphatase activity over a wide range of phosphosugars substrates (pH optima at 7.0 and *K*_m_ in the range of 3.6–7.7 mM). AtSgpp promiscuity is preferentially detectable on d-ribose-5-phosphate, 2-deoxy-d-ribose-5-phosphate, 2-deoxy-d-glucose-6-phosphate, d-mannose-6-phosphate, d-fructose-1-phosphate, d-glucose-6-phosphate, dl-glycerol-3-phosphate, and d-fructose-6-phosphate, as substrates. *AtSgpp* is ubiquitously expressed throughout development in most plant organs, mainly in sepal and guard cell. Interestingly, expression is affected by abiotic and biotic stresses, being the greatest under Pi starvation and cyclopentenone oxylipins induction. Based on both, substrate lax specificity and gene expression, the physiological function of AtSgpp in housekeeping detoxification, modulation of sugar-phosphate balance and Pi homeostasis, is provisionally assigned.

## Introduction

The haloacid dehalogenase-like hydrolase (HAD) superfamily is a large group of proteins with diverse substrate specificity whose members, despite the family name, are involved not only in the enzymatic cleavage by nucleophilic substitution of carbon–halogen bonds (C–halogen), but also in a variety of hydrolytic enzyme activities including phosphatase (CO–P), phosphonatase (C–P) and phosphoglucomutase (CO–P hydrolysis and intramolecular phosphoryl transfer) reactions (Koonin and Tatusov 1994; Allen and Dunaway-Mariano 2004).

Within the HAD superfamily, the wide family of magnesium-dependent acid phosphatases and phosphomutases is characterized by an amino-terminal conserved Asp as the nucleophile (Collet et al. 1998; Selengut 2001). All members share the α/β core domain catalytic scaffold, with the active site formed by four loops containing highly conserved sequence motifs (loops 1, DxD; 2, T/S; 3, K/R; 4, E/DD, GDxxxD, or GDxxxxD), spatially arranged around a single binding cleft, that position substrate–cofactor binding and catalytic residues that are involved in the core chemistry (Burroughs et al. 2006). Many of them also possess a smaller cap domain, linked to the core by two hinge-like solvated peptide linkers, which acts as a dynamic rigid lid over the core active site. Although its primary function might be active-site desolvation, the cap domain contains the helix–loop–helix (loop 5) with a stringently conserved Gly flanked by residues whose side chains contribute to the catalytic site formed at the domain–domain interface. These residues are responsible for the chemistry diversification within the family and provide substrate specificity (Allen and Dunaway-Mariano 2004; Lahiri et al. 2004). Thus, the elements involved in substrate-recognition and chemical-catalysis are located separately; substrate recognition is delegated to cap residues, whereas phosphoryl transfer is mediated by residues located deep inside the core cleft (Wang et al. 2002; Lu et al. 2005, 2008).

Based on the presence, topology and location of the cap domain, the HAD superfamily is divided into three subfamilies (I, IIA-IIB and III) (Morais et al. 2000; Zhang et al. 2002; Tremblay et al. 2006). In subfamily I, the small α-helical-bundle cap is inserted between loops 1 and 2 in the core domain, whereas in subfamily II the larger β-sandwich is placed between loops 2 and 3; the third group contains no insertion (Selengut and Levine 2000; Shin et al. 2003). Unlike the core catalytic domain, the cap has undergone extensive evolutionary diversity in substrate exploration during HADs evolution (Burroughs et al. 2006). While the α/β Rossmann core active sites are superimposable, the architecture of the cap domain differs even between the closest structural homologs (Rao et al. 2006).

In HAD phosphatases, after substrate bounding the enzyme active site is closed and Mg^2+^ cofactor interacts with the negatively charged phosphate, preparing it for nucleophilic attack by the first conserved Asp, forming an intermediate acyl-phosphate with the carboxyl group. Then, the enzyme opens again allowing the escape of the leaving group and the entry of solvent; water is deprotonated by the second Asp, hydrolyzing the acyl-phosphate intermediate and returning the enzyme to the native status (Burroughs et al. 2006). In phosphohydrolases, the Asp nucleophile is located on loop 1, loop 2 positions a conserved Ser/Thr that binds the substrate phosphoryl group, whereas loop 3 positions a conserved Arg/Lys that orients and shields charge in the Asp nucleophile and the phosphoryl group and, finally, loop 4 positions two or three Asp residues that bind the Mg^2+^ cofactor (Lu et al. 2005).

Due to their sequence divergence, only the highly conserved catalytic motifs and the similar folds and active-site structures allow identification between members of the HADs (Morais et al. 2000). However, the catalyzed reaction and substrate specificity are difficult to predict and have to be determined empirically (Kuznetsova et al. 2006).

In an earlier work, we cloned and characterized two *Arabidopsis thaliana* isoforms AtGpp1 (At4g25840) and AtGpp2 (At5g57440) of the dl-glycerol-3-phosphatase, involved in plant glycerol metabolism (Caparrós-Martín et al. 2007). The analysis of the sequence indicates that AtGpp1 and AtGpp2 phosphatase are members of the HAD haloacid dehalogenase hydrolase superfamily. AtGpp1 and AtGpp2 show high homology with the yeast phosphatases GPP1 and GPP2 (Norbeck et al. 1996), which have a high specificity for dl-glycerol-3-phosphate, as well as with DOG1 and DOG2 that dephosphorylate 2-deoxy-d-glucose-6-phosphate (Rández-Gil et al. 1995).

The *Arabidopsis* genome contains homologous loci, other than AtGpp1 and AtGpp2, with similar scores and general function predicted as phosphatase/phosphohexomutase (unknown At2g38740, putative At4g39970, catalytic/hydrolase At3g48420 and catalytic/hydrolase/phosphoglycolate phosphatase At2g33255). Attention was focused on these HAD superfamily-encoded loci, sharing significant similarity, presuming a related sequence-based assignment of activity on targeting substrates with similar structural characteristics, in *Arabidopsis*.

Following a screening approach, the four purified proteins were tested for phosphatase activity against a set of sugar phosphoesters. Pi release was only detected in assays with the product of locus *At2g38740* so-called AtSgpp. Structural prediction and the chemistry analysis reveal AtSgpp as a typical phosphomonoesterase of subclass I (C1-type cap). At odds with other subfamily I representatives such as phosphonatase, phosphoserine phosphatase, 2,3-diketo-l-phospho-5-thiomethylpentane phosphatase, 2-deoxy-d-glucose-6-phosphate phosphatase, and glycerolphosphate phosphatase, rather than being substrate specific, AtSgpp shows a broad-range sugar phosphate phosphatase activity. Curiously enough, similar specificity has been ascribed to the *Bacteroides thetaiotaomicron BT4131* gene (Lu et al. 2005). BT4131 enzyme is member of the haloalkanoate dehalogenase superfamily (subfamily IIB, C2B-type cap), with a tentatively assigned physiological function in chitin metabolism. The expected structure, specificity and kinetics of plant AtSgpp chemistry were analyzed and compared with those of bacterial BT4131.

## Materials and methods

### Materials

The materials used for cloning were: pMAL-c2X vector and *Escherichia coli* TB1 host for expression (New England Biolabs, Hitchin, Hertfordshire, UK), First Strand DNA synthesis kit for reverse transcriptase PCR (Roche Applied Science, Mannheim, Germany), REDTaq DNA polymerase (Sigma, St. Louis, MO, USA), oligonucleotides (Sigma-Genosys, Gillingham, Dorset, UK) and pBluescript SK+ vector (StrataGene, Kirkland, WA, USA). The RNA extraction was achieved with GenElute mammalian total RNA kit RTN70/TriReagent T9424 (Sigma, St. Louis, MO, USA). The pSBETa helper vector was constructed at the Max-Planck Institut (Köln, Germany) (Schenk et al. 1995) and the total RNA from siliqua was extracted as in Vicient and Delseny (1999).

### Plant material, growth conditions and stress treatments

*Arabidopsis thaliana* ecotype Columbia (Lehle Seeds, Round Rock, TX, USA) was grown in the greenhouse at 25 °C for 8 h in the dark and 16 h in light. For seedling stress assays, wild-type (WT) and transgenic surface-sterilized *Arabidopsis* seeds were sown in Petri dishes containing 3 ml MSS medium [MS (Murashige and Skoog 1962) M5524 (Sigma) + 1 % Agargel™ A-3301 (Sigma) + 3 % sucrose (Merck, Darmstadt, Germany)], MSS + 100 mM NaCl (Panreac, Barcelona, Spain) or 15 mM LiCl (Panreac) for salt stress, MSS + 200 mM sorbitol (Sigma) for osmotic stress, MSS + 5 mM H_2_O_2_ (Panreac) or 1 μM methyl viologen (Sigma) for oxidative stress. Seedlings were grown for 12 days at 25 °C under fluorescent light, 8 h dark and 16 h light.

### Genome, sequence and structural analysis

Comparative genomics were performed using programs such as BLAST (Altschul et al. 1990) and data bank resources from the NCBI. Protein domain families were generated with the ProDom program from the Swiss-Pro and TrEMBL sequence databases (Corpet et al. 2000). CLUSTAL W was used for the progressive multiple sequence alignment (Higgins et al. 1994). GENEVESTIGATOR *Arabidopsis* microarray database was utilized for the expression analysis (Zimmermann et al. 2004). 3D models of the protein have been built using the ESyPred3D web server (Lambert et al. 2002).

### cDNA cloning

Using peptide sequence motifs shared between the yeast dl-glycerol-3-phosphatases (Norbeck et al. 1996), virtual clones were isolated by BLAST (Altschul et al. 1990) search screening from the predicted conceptual translated proteins of the *A. thaliana* genomic library. The corresponding homologous genes were cloned by reverse PCR using leaf total RNA as template for the first strand cDNA synthesis together with an oligo(dT) primer and avian myeloblastosis virus reverse transcriptase. The first strand cDNA template was PCR amplified using REDTaq DNA polymerase and the 5′-forward and 3′-reverse gene-specific adapted primers 5′-CGAGGAATTCATGAATGGCTTCTCTGATCTTAATCC-3′/5′-CCGGGTCGACTTAAGACTTGTTATCAAGTTCTTCC-3′; 5′-CGAGGAATTCATGGCGGTTTCTTGCAACCACTCTGC-3′/5′CCGGGTCGACTTAAGCTGCAGTGACTATTGTTTGAAGC-3′; 5′-CGAGGAATTCATGGCCACTGTGAAAATCTCTCTTTCC-3′/5′-CCGGGTCGACTTAACTAACGAACTGTTTCCGGAG-3′; and 5′-CGAGGAATTCATGGCGAATTTAACGACGAACGC-3′/5′-CCGGGTCGACCTACGGGTTCAGGTCGAAGTTCG-3′ for At2g38740, At4g39970, At3g48420 and At2g33255, respectively. The PCR products were cloned as 735, 956, 960 or 675 bp *Eco*RI/*Sal*I fragment into the pBluescript SK + vector. Sequencing of the pBluescript SK + clones revealed that the sequence of the proteins is the same as that published in the Gen-Bank™. The cDNAs, containing the entire gene coding region, were subcloned using *Eco*RI/*Sal*I into the pMAL-c2X expression vector and transformed into the expression strain *E. coli* TB1 for recombinant protein production. The cloning site used in the pMAL-c2X polylinker (located downstream of the malE gene), adding vector-encoded residues Ile-Ser-Glu-Phe fused between the factor Xa cleavage site and the NH_2_-terminal methionine residue of the cloned proteins. To improve the expression of the eukaryotic genes in the *E. coli* system, *E. coli* TB1 cells were co-transformed with pMAL-c2X At2g38740, pMAL-c2X At4g39970, pMAL-c2X At3g48420 or pMAL-c2X At2g33255 and, in each case, the helper plasmid pSBETa. The positive co-transformed colonies were selected on 200 μg/ml ampicillin (Sigma) and 100 μg/ml kanamycin (Sigma).

### Purification of recombinant proteins

Selected co-transformed *E. coli* strains, containing fusion plasmid pMAL-c2X At2g38740, pMAL-c2X At4g39970, pMAL-c2X At3g48420 or pMAL-c2X At2g33255 and, in each case, the helper pSBETa, were gown at 37 °C to 2 × 10^8^ cells/ml (*A*_600_ ~0.5) in 1 l of rich broth + glucose and ampicillin + kanamycin (10 g tryptone, 5 g yeast extract, 5 g NaCl, 2 g glucose, autoclave; add sterile 200 μg/ml ampicillin and 100 μg/ml kanamycin), induced with 1 mM isopropyl-β-d-thiogalactoside (IPTG) (Ambion, Austin, TX, USA) and harvested 2 h after induction. Fusion proteins were released from the harvested cells by sonication in column buffer (20 mM Tris–HCl pH 7.4, 200 mM NaCl, and 1 mM EDTA), collected after elution from the amylose resin (New England Biolabs) with column buffer + 10 mM maltose (Sigma) and the concentration determined by the Bradford method (1976). Proteins were separated by SDS-PAGE electrophoresis in 12 % polyacrylamide gels (Schagger and von Jagow 1987) using prestained molecular weight standards (New England Biolabs).

### Activity assays

The biochemical characterisation of the purified enzymes was assayed as previously described by Sussman and Avron (1981). The reaction mixture contained 20 mM Tris–HCl pH 7.0, 5 mM MgCl_2_, and 10 mM d-ribose-5-phosphate, 2-deoxy-d-ribose-5-phosphate, 2-deoxy-d-glucose-6-phosphate, d-mannose-6-phosphate, d-fructose-1-phosphate, d-glucose-6-phosphate, dl-glycerol-3-phosphate, d-fructose-6-phosphate, α-d-glucose-1-phosphate, α-d-mannose-1-phosphate, d-fructose-1,6-bisphosphate, α-d-glucose-1,6-bisphosphate or d-erythrose-4-phosphate (R7750, D3126, D8875, M6876, F1127, G7250, G2138, F1502, G9380, M1755, F4757, G5750, E0377, respectively) (Sigma) in a total volume of 1 ml. Approximately 25 μg/ml of purified protein was used in the enzymatic reactions. After 15, 30 and 60 min of incubation with the enzyme, samples of 0.3 ml were withdrawn and added to 0.7 ml phosphate determination mixture. The released inorganic phosphate was determined according to Ames (1966) and the reaction rate was calculated in relation to the amount of enzyme and time. Different substrate concentrations (0.5–32 mM) were used to receive the Michaelis–Menten kinetic parameters *K*_m_ and *V*_max_. The *k*_cat_ was calculated from the equation: *k*_cat_ = *V*_max_/[*E*] ([*E*] enzyme concentration in molar). To demonstrate the influence of the pH, reaction mixtures with the pH range of 2–10 were used; the experiments were done at least twice with values differing not significantly.

### Northern blots and hybridization

Northern analyses were performed using approximately 20 mg of total RNA per track. Isolated DNA fragments were nick-translated in the presence of α-[^32^P]dCTP to be used as probes (Maniatis et al. 1982). Probe *AtSgpp* cDNA was a 735-bp *Eco*RI/*Sal*I fragment, which contains the complete *AtSgpp* coding region. Hybridization was performed in 3× SSC (saline sodium citrate), 0.05 % PVP, 0.05 Ficoll, 1 % SDS and 50 μg ml^−1^ ssDNA (salmon sperm DNA) (Sigma) at 55 °C. Filters were washed at high stringency (0.1× SSC and 0.5 % SDS) at 55 °C. The experiments were performed more than once and the data shown are representative.

## Results

### Isolation of *A. thaliana AtGpp*-homologous genes

In previous work, the complete sequence of the *Arabidopsis* genome (The Arabidopsis Genome Initiative 2000) was used for the identification of the two uncharacterised *A. thaliana* dl-glycerol-3-phosphatase genes. With the known budding yeast GPP1 and GPP2 protein sequences and using the BLAST program (Altschul et al. 1990) two putative *A. thaliana*, named *AtGpp1* and *AtGpp2*, were identified, cloned and characterized (Caparrós-Martín et al. 2007). Using a comparative genome approach, four additional homologous genes were detected, with similar scores and general function predicted as phosphatase/phosphohexomutase (unknown *At2g38740*, putative *At4g39970*, catalytic/hydrolase *At3g48420* and catalytic/hydrolase/phosphoglycolate phosphatase *At2g33255*). The virtual isolated genes were cloned by RT-PCR and sequenced.

### Gene thin structure

In agreement with the NCBI reported annotations, loci *At2g38740*, *At4g39970*, *At3g48420* and *At2g33255* are encoded on *A. thaliana* chromosome 2, 4, 3 and 2 as 1,786, 1,751, 1,808 and 1,563-bp length genes with 3, 9, 7 and 4 introns, respectively, with unspliced 5′ and 3′-untranslated regions. The At2g38740, At4g39970, At3g48420 and At2g33255 proteins have 244, 316, 319 and 245 amino acids, a deduced Mw of 26.7, 34.7, 34.3 and 27.5 kDa and pI of 4.6, 5.2, 8.2 and 6.1, respectively. At2g38740 does not have any predicted signal to mitochondrial or chloroplast targeting, while for At4g39970, At3g48420 and At2g33255 its location is expected to be in the chloroplast.

### Sequence comparisons

**Fig. 1:**
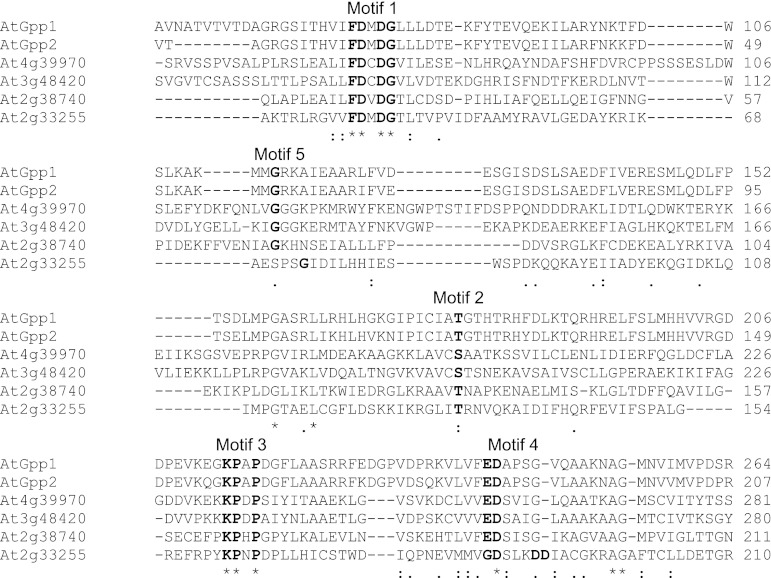
AtGpp alignment with its homologous proteins. The amino acid sequence of the *A. thaliana* dl-glycerol-3-phosphatases AtGpp1 and AtGpp2 was compared with their homologous representatives from *A. thaliana* At2g38740, At4g39970, At3g48420 and At2g33255. Residues that are identical or similar to AtGpp1 and AtGpp2 are *highlighted with asterisks or dots*, respectively, and the shared motifs are depicted in *bold*

The four plant proteins show significant similarity (28–39 %, sequence identity of 18–23 %) to *A. thaliana* dl-glycerol-3-phosphatases AtGpp1 and AtGpp2 (Fig. 1). This homology is ubiquitously distributed although, as it would be expected among HAD members, the greatest similarity occurs at the four loops forming the catalytic scaffold of the active site platform framed by the core domain (Zhang et al. 2004; Lu et al. 2005), particularly in the highly conserved sequence motifs by which family members are recognized, contained in loops 1–4 (1, FDxDG; 2, xT/Sx; 3, KPxP; 4, ED, or GDxxxDD), that positions substrate–cofactor binding and catalytic residues that are involved in the core chemistry (Burroughs et al. 2006). Fewer sequence homologies are shared at the predicted cap domain substrate recognition loop 5, responsible for the chemical diversification within the family, apart from the stringently conserved loop marker Gly (G), flanked by Lys/Arg (K/R) and residues, whose side chains contribute to the catalytic site, probably operating in domain–domain binding, active-site desolvation and/or catalysis (Lahiri et al. 2004), that must provide the signature-based substrate specificity.

### Protein expression, purification and substrate screening

**Fig. 2:**
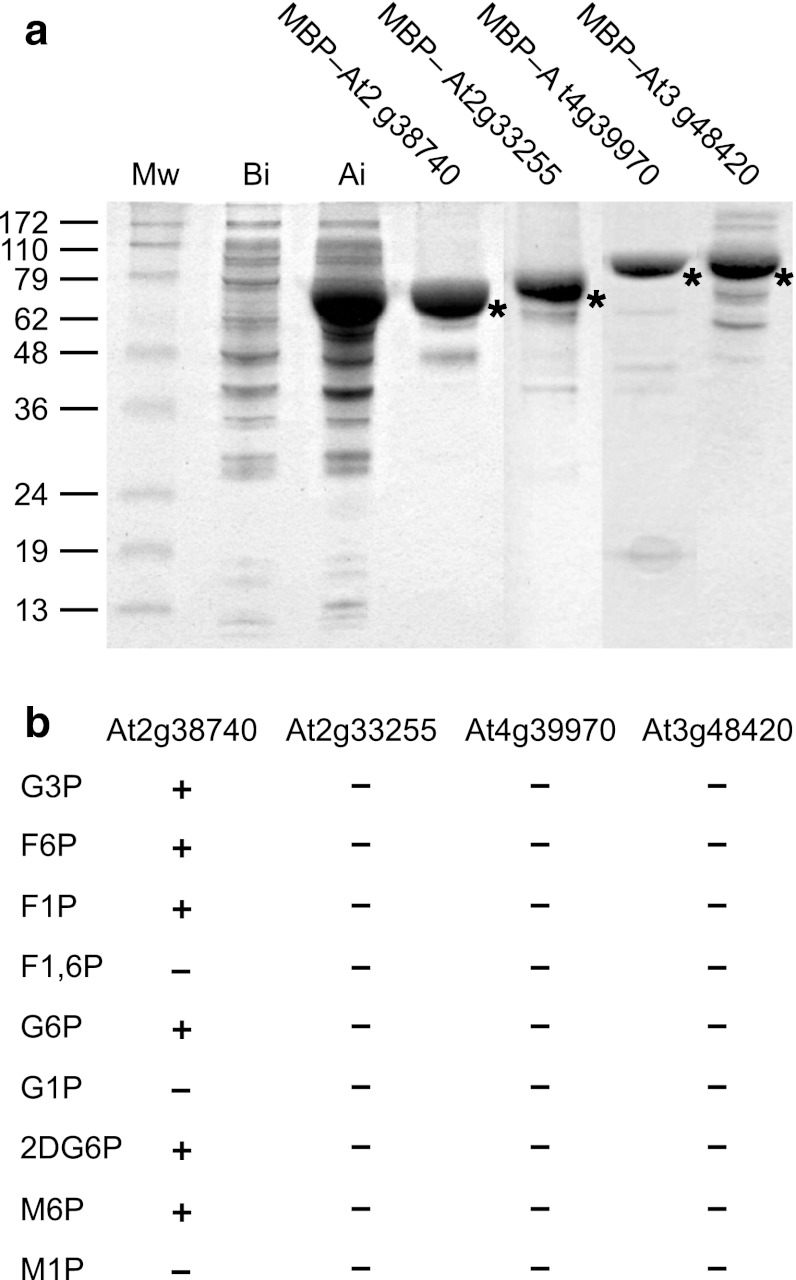
Purification and hydrolytic activity of AtGpp homologues. **a**, **b** SDS-PAGE of purified MBP-fusion proteins by amylose affinity chromatography. Prestained molecular weight marker in kDa (*Mw*); MBP-At2g38740 before induction (*Bi*); MBP-At2g38740 after induction (*Ai*); partially purified MBP-proteins: At2g38740, At2g33255, At4g39970, and At3g48420 (**a**). Hydrolytic activity of the AtGpp homologous At2g38740, At4g39970, At3g48420, and At2g33255 on dl-glycerol-3-phosphate (*G3P*), d-fructose-6-phosphate (*F6P*), d-fructose-1-phosphate (*F1P*), d-fructose-1,6-bisphosphate (*F1,6P*), d-glucose-6-phosphate (*G6P*), α-d-glucose-1-phosphate (*G1P*), 2-deoxy-d-glucose-6-phosphate (*2DG6P*), d-mannose-6-phosphate (*M6P*), and α-d-mannose-1-phosphate (*M1P*) (**b**). Purified MBP fusion proteins are indicated by *asterisks*. (+) Positive and (−) negative hydrolysis

To examine the phosphatase activity, the AtGpp homologous At2g38740, At4g39970, At3g48420 and At2g33255 (26.7, 34.7, 34.3 and 27.5 kDa, respectively) were expressed as MBP (42.7 kDa) fusion proteins, purified from the corresponding *E. coli* clones, by amylose affinity chromatography, and their phosphatase activity determined in the presence of Mg^2+^ ions (Fig. 2).

Presuming a connected sequence-based assignment of function on targeting substrates with similar structural characteristics, the four purified proteins (Fig. 2a) were investigated for dl-glycerol-3-phosphatase activity and against a set of related phosphoesters such as d-fructose-6-phosphate, d-fructose-1-phosphate, d-fructose-1,6-bisphosphate, d-glucose-6-phosphate, α-d-glucose-1-phosphate, 2-deoxy-d-glucose-6-phosphate, d-mannose-6-phosphate, and α-d-mannose-1-phosphate. Pi release, from hydrolyzed substrates, was found in assays with MBP–At2g38740, whereas there was no hydrolytic activity detectable for MBP–At4g39970, MBP–At3g48420, or MBP–At2g33255 (Fig. 2b) even though their predicted cap domains are remarkably similar in fold (result not shown). Yet it cannot be discounted that the examined inactive proteins were expressed and purified in a functionally folded form. Table 1 represents the in vitro activity of purified At2g38740 on various organic phosphoesters. The plant enzyme, hereafter called *A. thaliana* phosphosugar phosphatase AtSgpp, shows a broad-range sugar phosphate phosphatase affinity. The activity is preferentially detectable on d-ribose-5-phosphate, 2-deoxy-d-ribose-5-phosphate, 2-deoxy-d-glucose-6-phosphate, and d-mannose-6-phosphate; lower activity is observed on d-fructose-1-phosphate, d-glucose-6-phosphate, dl-glycerol-3-phosphate, and d-fructose-6-phosphate. The phosphorylated compounds α-d-glucose-1-phosphate, α-d-mannose-1-phosphate, d-fructose-1,6-bisphosphate, α-d-glucose-1,6-bisphosphate, and d-erythrose-4-phosphate were also tested and showed no significant activity.

**Table 1:** Phosphatase activity of purified AtSgpp on various organic phosphoesters

Substrate	Relative activity
Ribose-5-phosphate	100
2-Deoxyribose-5-phosphate	93
2-Deoxyglucose-6-phosphate	91
Mannose-6-phosphate	85
Fructose-1-phosphate	77
Glucose-6-phosphate	70
dl-Glycerol-3-phosphate	55
Fructose-6-phosphate	51
Glucose-1-phosphate	–
Mannose-1-phosphate	–
Fructose-1,6-bisphosphate	–
Glucose-1,6-bisphosphate	–
Erythrose-4-phosphate	–

The reaction mixture contained: 20 mM Tris–HCI (pH 7.0), 5 mM MgCI_2_, 10 mM substrate (different phosphoesters) and 25 µg/ml of the purified protein. Reaction temperature: 32 °C. The enzyme activity with d-ribose-5-phosphate was set to 100

### Kinetic analysis

**Fig. 3:**
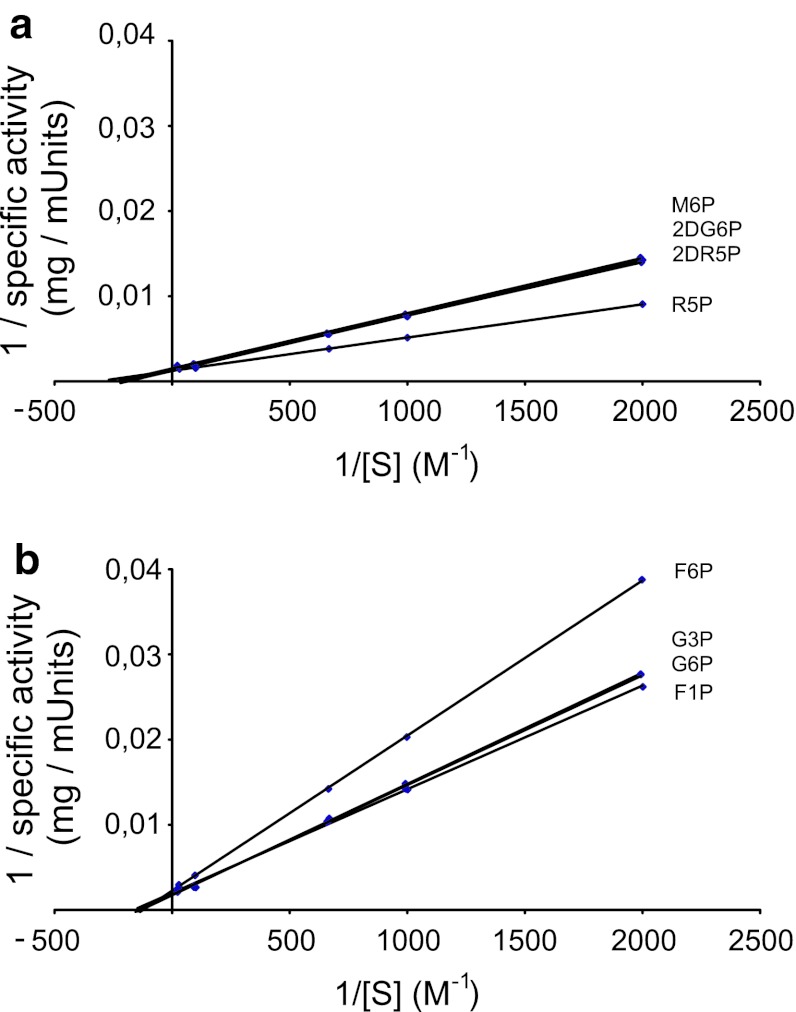
AtSgpp phosphatase activity profile. **a**, **b** The apparent *K*_m_ and *V*_max_ of the AtSgpp phosphatase for d-ribose-5-phosphate (R5P), 2-deoxy-d-ribose-5-phosphate (2DR5P), 2-deoxy-d-glucose-6-phosphate (2DG6P), d-mannose-6-phosphate (M6P) (**a**); for d-fructose-1-phosphate (F1P), d-glucose-6-phosphate (G6P), dl-glycerol-3-phosphate (G3P), d-fructose-6-phosphate (F6P) (**b**). The reaction mixture contained: 20 mM Tris–HCl (pH 7.0), 5 mM MgCl_2_, the indicated concentration of substrate, 25 μg/ml of AtSgpp. Reaction temperature: 32 °C

AtSgpp protein was further investigated for substrate specificity. The apparent *K*_m_ and *V*_max_ values of MBP–AtSgpp for phosphoesters/Mg^2+^ were determined by non-linear regression from spectrophotometric data (Fig. 3). The corresponding slopes of the Lineweaver–Burk plot fall into two differentiated categories, lower *K*_m_/*V*_max_ values are obtained for d-ribose-5-phosphate, 2-deoxy-d-ribose-5-phosphate, 2-deoxy-d-glucose-6-phosphate, and d-mannose-6-phosphate (Fig. 3a), than for those of d-fructose-1-phosphate, d-glucose-6-phosphate, dl-glycerol-3-phosphate, and d-fructose-6-phosphate (Fig. 3b).

The catalytic hydrolysis occurs with inconspicuous specificity and efficiency; the values of the substrate specificity constant *k*cat/*K*_m_ for d-ribose-5-phosphate, 2-deoxy-d-ribose-5-phosphate, 2-deoxy-d-glucose-6-phosphate, d-mannose-6-phosphate, d-fructose-1-phosphate, d-glucose-6-phosphate, dl-glycerol-3-phosphate, and d-fructose-6-phosphate are in the range of 2.5–10.7 × 10^3^ M^−1^ s^−1^, being the highest *k*_cat_/*K*_m_ value for d-ribose-5-phosphate (10.7 × 10^3^ M^−1^ s^−1^) and the lowest for d-fructose-6-phosphate (2.5 × 10^3^ M^−1^ s^−1^) (Table 2).

**Table 2:** Kinetics parameters of the phosphatase activity of purified AtSgpp on various organic phosphomonoesters

Substrate	*V*_max_ (µmol s^−1^)	*K*_m_ (M)	*k*_cat_ (s^−1^)	*k*_cat_/*K*_m_ (M^−1^ s^−1^)
d-Ribose-5-phosphate	(13.8 ± 0.5) × 10^−3^	(3.6 ± 0.1) × 10^−3^	(3.9 ± 0.3) × 10	10.7 × 10^3^
2-Deoxy-d-ribose-5-phosphate	(11.9 ± 0.3) × 10^−3^	(4.5 ± 0.3) × 10^−3^	(3.3 ± 0.2) × 10	7.3 × 10^3^
2-Deoxy-d-glucose-6-phosphate	(11.8 ± 0.6) × 10^−3^	(4.6 ± 0.5) × 10^−3^	(3.3 ± 0.1) × 10	7.2 × 10^3^
d-Mannose-6-phosphate	(11.6 ± 0.4) × 10^−3^	(4.9 ± 0.6) × 10^−3^	(3.2 ± 0.6) × 10	6.7 × 10^3^
d-Fructose-1-phosphate	(9.2 ± 0.3) × 10^−3^	(6.9 ± 0.1) × 10^−3^	(2.6 ± 0.4) × 10	3.7 × 10^3^
d-Glucose-6-phosphate	(8.7 ± 0.2) × 10^−3^	(7.1 ± 0.3) × 10^−3^	(2.4 ± 0.5) × 10	3.4 × 10^3^
dl-Glycerol-3-phosphate	(8.3 ± 0.2) × 10^−3^	(7.3 ± 0.2) × 10^−3^	(2.3 ± 0.3) × 10	3.1 × 10^3^
d-Fructose-6-phosphate	(6.9 ± 0.1) × 10^−3^	(7.7 ± 0.4) × 10^−3^	(1.9 ± 0.2) × 10	2.5 × 10^3^

The reaction mixture contained: 20 mM Tris–HCI (pH 7.0), 5 mM MgCI_2_, 10 mM substrate (different phosphomonoesters) and 25 µg/ml of the purified protein. Reaction temperature: 32 °C

### pH rate profile determinations

**Fig. 4:**
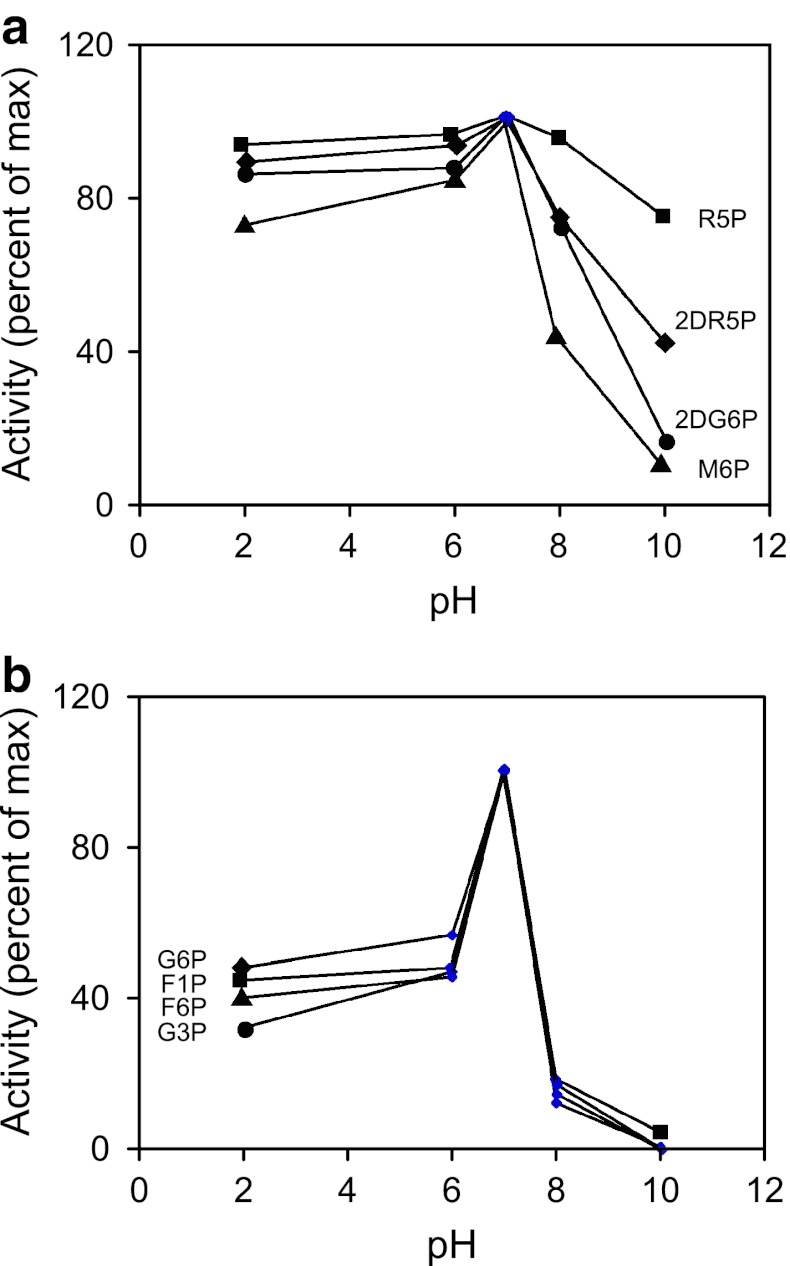
AtSgpp pH dependence. **a**, **b** Influence of pH on the phosphatase activity of AtSgpp for d-ribose-5-phosphate (R5P, *solid square*), 2-deoxy-d-ribose-5-phosphate (2DR5P, *solid diamond*), 2-deoxy-d-glucose-6-phosphate (2DG6P, *solid circle*), d-mannose-6-phosphate (M6P, *solid triangle*) (**a**); d-fructose-1-phosphate (F1P, *solid square*), d-glucose-6-phosphate (G6P, *solid diamond*), dl-glycerol-3-phosphate (G3P, *solid circle*), d-fructose-6-phosphate (F6P, *solid triangle*) (**b**). The reaction mixture contained: 20 mM Tris–HCl (the indicated pH), 5 mM MgCl_2_, 10 mM substrate, 25 μg/ml of AtSgpp. Reaction temperature: 32 °C

Like most HAD phosphatases, AtSgpp developed optimal activity toward natural substrates at neutral pH 7.0 (Fig. 4). Interestingly, the pH dependence of MBP–AtSgpp catalysis on d-ribose-5-phosphate, 2-deoxy-d-ribose-5-phosphate, 2-deoxy-d-glucose-6-phosphate, and d-mannose-6-phosphate (Fig. 4a), again differs from those of d-fructose-1-phosphate, d-glucose-6-phosphate, dl-glycerol-3-phosphate, and d-fructose-6-phosphate (Fig. 4b). It is striking that the MBP–AtSgpp targeting per d-ribose-5-phosphate does not significantly change over the entire pH range, whilst it decreases as pH increases in the case of 2-deoxy-d-ribose-5-phosphate, 2-deoxy-d-glucose-6-phosphate, and d-mannose-6-phosphate. However, MBP–AtSgpp affinity abruptly drops, following a graph bell-shaped, on d-fructose-1-phosphate, d-glucose-6-phosphate, dl-glycerol-3-phosphate, and d-fructose-6-phosphate, when reaching an acidic or basic pH. This contrasting behavior may result from pH-dependent differences, in active site desolvation and conformational entropy, upon substrate binding and the induced cap closure.

### *AtSgpp* gene expression

**Fig. 5:**
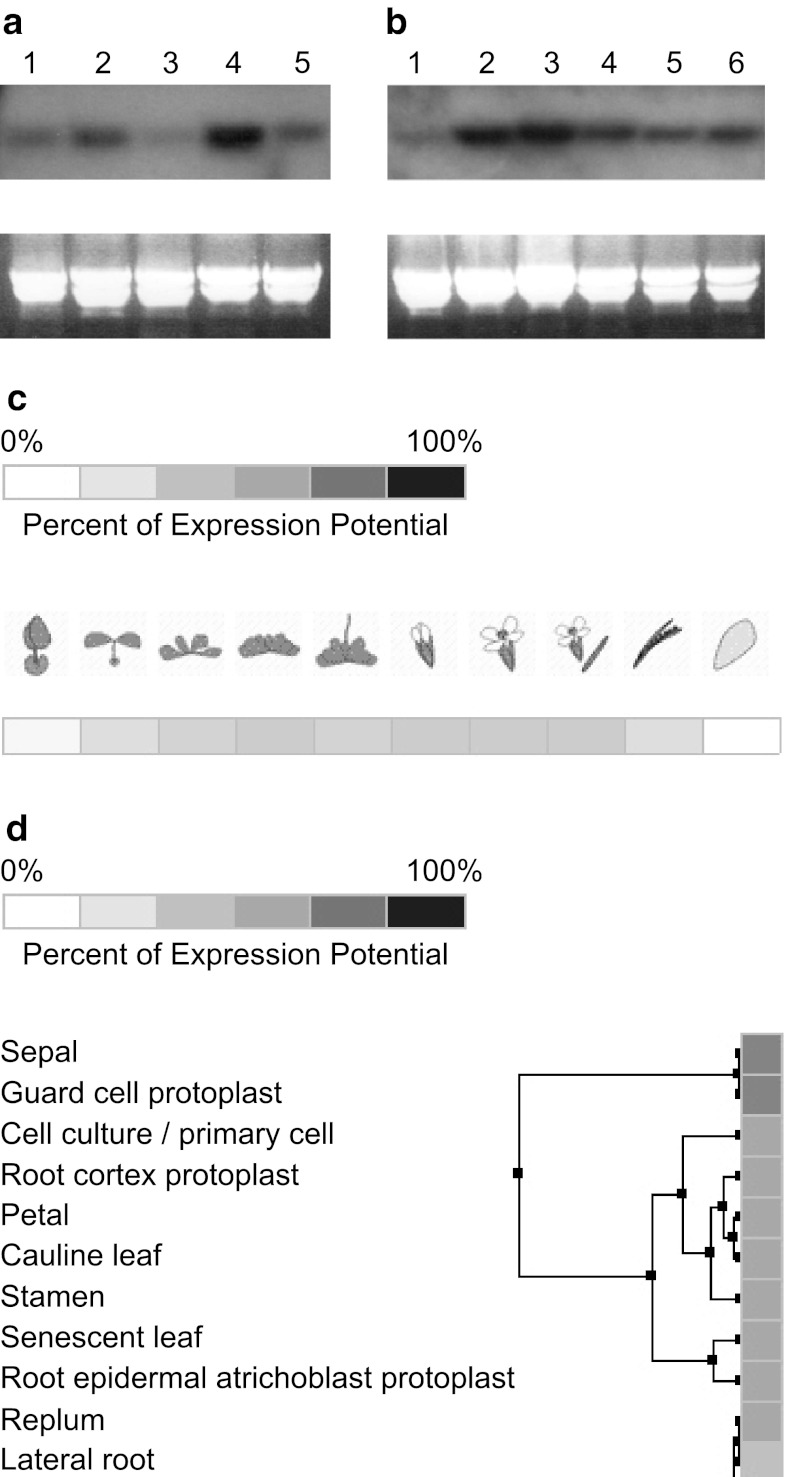
Expression of *AtSgpp* mRNA during development and under stress treatment. **a**, **b** Northern blot of *Arabidopsis* RNA probed with radiolabelled *AtSgpp* cDNA. **c**, **d** *AtSgpp* pattern of expression from the microarrays data bank Genevestigator. **a** From different tissues: *1* root, *2* shoot, *3* leaves, *4* flowers, *5* siliqua. **b** From 12-day-old seedling cultivated on MSS medium: *1* control, *2* MSS + 100 mM NaCl, *3* MSS + 200 mM sorbitol, *4* MSS + 15 mM LiCl, *5* MSS + 5 mM H_2_O_2_ and *6* MSS + 1 μM methyl viologen. In Northern blots, ethidium bromide-stained RNA was used as loading control. **c** Expression profile over time throughout the live cycle of the *Arabidopsis*. **d** Partial hierarchical clustering displaying the level of the anatomical expression across of tissue types

To gain insight into the physiological importance of the AtSgpp hydrolytic activity in plants, the expression of the *A. thaliana* *AtSgpp* gene was analyzed, by Northern hybridization, in different tissues and under stress conditions (Fig. 5). In all blot experiments a band was detected corresponding in size to the *AtSgpp* transcripts. The *AtSgpp* pattern of expression was examined in roots, shoots, leaves, flowers and developing siliqua (Fig. 5a). Transcripts of the gene were detectable on northern blots from all of these organs. High expression was found in flowers, pointing to a prominent role of this gene during floral development. *AtSgpp* mRNA accumulation was also observed in shoots, siliqua, roots and the lowest in leaves. *AtSgpp* expression was also examined in seedling subjected to salt (100 mM NaCl and 15 mM LiCl), osmotic (200 mM sorbitol) or oxidative (5 mM H_2_O_2_ and 1 μM methyl viologen) stresses (Fig. 5b). *AtSgpp* expression seems to be affected in seedlings that undergo all these abiotic stresses.

More detailed information about the *AtSgpp* pattern of expression was obtained from the microarrays data bank Genevestigator (Zimmermann et al. 2004). Expression during development is medium, declining to low with senescence (Fig. 5c), while the anatomical expression is in most plant organs, leading to their hierarchical clustering in the sepals and the guard cell protoplasts (Fig. 5d). The quantitative transcriptomic analysis also shows that expression of locus *At2g38740* is perturbed by abiotic and biotic stresses, being up-regulated by cold, heat, oxidative, osmotic, salt, drought, hypoxia, genotoxic, and bounding, as well as in response to pathogens (not shown).

### Signature sequence substrate specificity loop 5

**Fig. 6:**
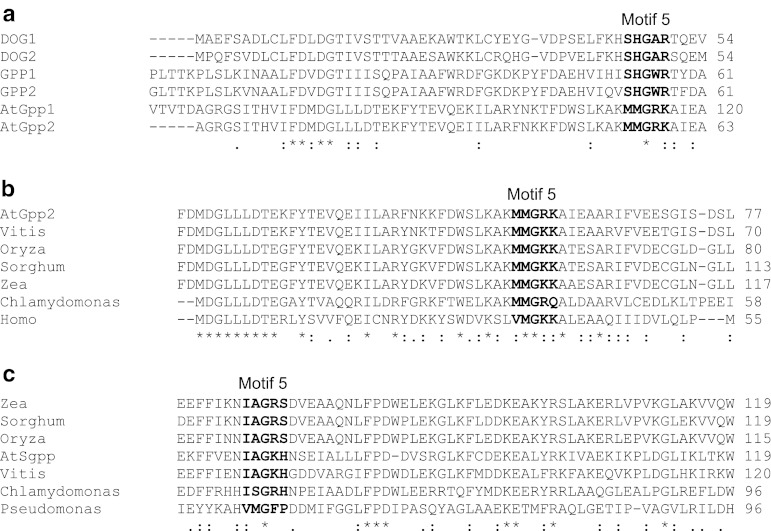
Comparison of the signature sequence motif 5. **a** The amino acid sequence of *A. thaliana* dl-glycerol-3-phosphatases AtGpp1 and AtGpp2 was compared with homologous representatives from *S. cerevisiae* dl-glycerol-3-phosphatases GPP1 and GPP2 and 2-deoxy-d-glucose-6-phosphatases DOG1 and DOG2. **b**, **c** *Arabidopsis* dl-glycerol-3-phosphatase AtGpp2 and phosphosugar phosphatase AtSgpp were also compared with orthologous candidates from other genus by NCBI BLink: *Vitis* (CBI17840), *Oryza* (BAD05444), *Sorghum* (EES14772), *Zea* (ACF85466), *Chlamydomonas* (EDP00037), and *Homo* (AAH12494) (**b**) and *Vitis* (CBI33932), *Oryza* (BAD36300), *Sorghum* (EER89058), *Zea* (ACF83536), *Chlamydomonas* (EDP04776), and *Pseudomonas* (EGH95925) (**c**), respectively. Conserved residues are labeled with *asterisks or dots* and the putative shared motifs in *bold*. In the absence of a three-dimensional structure, cap domain-derived active site residues were identified by the conjoined information from predicted 3D models, the boundaries of the cap sequence segment defined by motifs 1 and 2, and the conserved Gly (G) that identify the loop 5 motif within this segment

While the catalytic core is superimposable, the cap domain has evolved through the evolution, remaining responsible of targeting diversification among members of the subfamily. The cap contains the so-called substrate specificity loop 5 that provides the signature sequence motif 5 which, in the closed conformation of the enzyme, defines the active site and its specific chemistry. Core sequence motifs 1 and 2 determine the boundaries of the cap sequence segment in subfamily I, and the conserved Gly identifies the loop 5 motif within it, allowing detection of cap domain-derived active site residues, in the absence of a three-dimensional structure (Lahiri et al. 2004). Using this effective tool for function assignment together with their predicted structure, the substrate specificity loop 5 from known HAD phosphohydrolases GPP (Norbeck et al. 1996), DOG (Rández-Gil et al. 1995), AtGpp (Caparrós-Martín et al. 2007), AtSgpp and unknown homologous representatives, were compared (Fig. 6).

In baker’s yeast subclass I dl-glycerol-3-phosphatases GPP1 and GPP2 and 2-deoxy-d-glucose-6-phosphatases DOG1 and DOG2, their putative shared motif 5 (SHGW/AR) presents a single shift of Trp (W56 in GPP1 and GPP2) per Ala (A49 in DOG1 and DOG2) which could impart substrate specificity, while between yeast GPP1 and GPP2 and its plant counterparts AtGpp1 and AtGpp2, just the central Gly (G55 in GPP1 and GPP2 and G57 in AtGpp2), essential in determining the loop 5 conformation and flexibility (Lahiri et al. 2004), and surrounding Lys (R57 in GPP1 and GPP2 and R58 in AtGpp2) are conserved (Fig. 6a). By contrast, the motif 5 is well preserved between genuses within the kingdom itself (Fig. 6b, c). Lack of shared homology is also appreciated among phosphosugar phosphatases, subfamily IIB bacterial BT4131 and subclass I plant AtSgpp, despite their analogous catalysis and substrate specificity (not shown).

## Discussion

The objective of this study was to search for the sequence–function relationship, which drives the substrate discrimination between HAD phosphohydrolases subfamily members. Application of general enzymatic screenings and substrate profiling is a useful tool to discover new enzymes (Kuznetsova et al. 2005); likewise, the substrate prediction of unknown HAD hydrolases and their confirmed function, by applying the predicted substrates to the purified protein, has been an elegant strategy that has been successfully carried out (Lu et al. 2005). Following sequence–function analysis, as those used in the characterization of *Arabidopsis* dl-glycerol-3-phosphatases AtGpp1 and AtGpp2 (Caparrós-Martín et al. 2007), the physiological substrate specificity of the closest AtGpp homologues At2g38740, At4g39970, At3g48420, and At2g33255 was analyzed. Rather than for their genomic context, loci were chosen for the remarkable shared similarity of the encoding genes. In the spirit of the initial strategy it was to contribute to the hypothesis that might be possible to infer guidelines for function assignment within the HAD family, based on the sequence data alone (Lahiri et al. 2004); abounding into the syllogism that, if the cap domain defines substrate specificity and the physiological substrate determines the biochemical function, logically the relationship between cap domain and substrate structure is the essential clue to the sequence-based assignment of its function (Tremblay et al. 2006).

The AtSgpp catalytic hydrolysis of cyclic sugars occurs with inconspicuous specificity and efficiency, *k*cat/*K*_m_ in the range of 2.5–10.7 × 10^3^ M^−1^ s^−1^. Such a modest catalysis and substrate promiscuity has been reported by the hexose phosphate phosphatase HPP (BT4131 from *Bacteroides thetaiotaomicron* VPI-5482), whose catalytic efficiency is low: *k*cat/*K*_m_ ~1 × 10^3^ M^−1^ s^−1^ compared to the 1 × 10^6^ to 1 × 10^8^ M^−1^ s^−1^ “gold-standard” for single-substrate enzymes involved in primary metabolism (Lu et al. 2005). Bacterial BT4131 is a member of the haloalkanoate dehalogenase superfamily (subfamily IIB) with a C2B-type cap domain, whilst the expected structure of the plant enzyme yet enrolls AtSgpp as a member of the subfamily I with the C1-type cap domain. Besides the lax specificity for the ring size and stereochemistry of the sugar phosphate, AtSgpp shares with BT4131 the ability to discriminate between isomers that do not accommodate into the active site solvent cage, such as d-glucose-1-phosphate or α-d-mannose-1-phosphate and, to a lesser extent, d-fructose-6-phosphate. As suggested for other HAD monophosphatases (Kuznetsova et al. 2006; Tremblay et al. 2006), the kinetic parameters of AtSgpp demonstrated an overlapping catabolic activity, with undefined boundaries on physiologically related substrates that, in this case, point to a role in the metabolic regulation of phosphosugars intermediates of the glycolysis and the pentose phosphate pathway. The activity of AtSgpp on 2-deoxy-d-glucose-6-phosphate also arouses interest. 2-deoxy-d-glucose is a non-metabolizable analog of glucose that becomes toxic after its phosphorylation to 2-deoxy-d-glucose-6-phosphate. So far, no plant analogous to the yeast *DOG1* and *DOG2* genes (Rández-Gil et al. 1995), which dephosphorylate 2-deoxyglucose-6-phosphate, has been found. However, *DOG2* transgenic over-expression in tobacco improves the tolerance to the deleterious effects of 2-deoxy-d-glucose on growth, chlorophyll content and the expression of genes related to photosynthesis (Cutanda 2003). In vitro phosphatase activity against 2-deoxyglucose-6-phosphate has also been observed in *E. coli* YniC (HAD1) protein; *E. coli* YniC-overproducing strain grew well in the presence of 2-deoxy-d-glucose, demonstrating that the 2-deoxy-d-glucose-6-phosphatase activity of YniC (HAD1), plays an important role for the resistance of *E. coli* cells to 2-deoxyglucose (Kuznetsova et al. 2006).

Substrate binding regulates, by the hinge motion of the solvated domain linkers, the conformational cap closed/open interconversion. In the cap-closed conformation, cap and core domain interfaced and residues from the cap substrate specificity loop enter the active site, participating in substrate binding and catalysis, whereas the cap-open conformation allows the access to the active site of the solvent, facilitating the release of the product (Morais et al. 2004). Similarly, as previously reported for subclass I phosphonatase catalysis (Zhang et al. 2002; Morais et al. 2004; Lahiri et al. 2006), one could also suggest that the substrate specificity loop of AtSgpp contributes the catalytic Lys (K71) residue to form a Schiff base with the substrate. Proton dissociation from the K71 in the cap domain versus protonation of His (H72) would be required for the AtSgpp cap–core domains closure. Together with the ionization of several enzyme and substrate groups, the loss of activity at low pH could be due to the protonation of H72 and at high pH to deprotonation of K71. Thus, extreme pH would modify the intramolecular proton-transfer, introducing a change in the electron density of the system and on the stability of the phosphoaspartate intermediate compound. This being the case, the pH dependency of the AtSgpp chemistry on d-ribose-5-phosphate/Mg^2+^ would reflect a narrower chemoselectivity in the active site than those conformed by the remaining ligands tested.

Similar pattern of *AtSgpp* gene expression, in all *Arabidopsis* organs, have been shown for dl-glycerol-3-phosphatase AtGpp (Caparrós-Martín et al. 2007), with transcripts particularly abundant in developing siliqua. Particularly interesting is the induced expression under nitrogen and iron deficiency and the abscisic acid-induced and reactive oxygen species-dependent expression in guard cell (Böhmer and Schroeder 2011). Foremost, the greatest expression is observed under Pi starvation and cyclopentenone oxylipins induction. Oxylipins induce the expression of genes related to detoxification, stress responses, and secondary metabolism, when accumulated in response to stress stimuli such as wounding and pathogen infection, what has been attributed to an increase in ROS (Mueller et al. 2008). Coincidentally, a housekeeping function has also been assigned to the hydrolyzing activity of AtCoase, a pyrophosphatase from *Arabidopsis thaliana* which cleaves coenzyme-A to 4′-phosphopantetheine and 3′,5′-adenosinediphosphate; the CoA cleaving enzyme, whose ubiquitous expression improves plant development, is a member of the Nudix hydrolases, pyrophosphatases that hydrolyze nucleoside diphosphates (Kupke et al. 2009).

Since the referred signature sequence motif 5 drives the substrate targeting, and consequently may be used as protein tagging, from the sequence motif comparisons, it would be worth considering that, within the same species a unique point mutation Trp/Ala (W/A) may be sufficient to discriminate between structural related substrates, pivoting the stereospecificity for dl-glycerol-3-phosphate or 2-deoxy-d-glucose-6-phosphate. In contrast, orthologous enzymes as dl-glycerol-3-phosphatases, from different kingdoms, differ in their patterns. Most intriguing if it fits, in HADs evolutionary divergence, is the ability of analogous Bacteria-BT4131 and Plantae-AtSgpp to perform similar function with dissimilar topology and location of the cap domain. Considering that, although both the I and II subfamilies act predominantly on small encapsulated substrates (Allen and Dunaway-Mariano 2004), in subfamily I the small α-helical-bundle C1-type cap is inserted between loops 1 and 2 of the core domain, whereas in subfamily IIB the larger β-sandwich C2B-type is between loops 2 and 3 (Selengut and Levine 2000; Shin et al. 2003); moreover, BT4131 uses two substrate specificity loops in substrate recognition (Lu et al. 2005).

In conclusion, this paper reports the enzymatic characterization of *A. thaliana* locus *At2g38740*. Rescued from the HAD unknown members and hereafter named as phosphosugar phosphatase AtSgpp, its expected structure lists the enzyme as being a member of the HAD subfamily I C1-type cap domain. Extensive substrate screening reveals that AtSgpp presents substrate promiscuity, with broad-range sugar phosphate phosphatase activity, preferentially detectable on d-ribose-5-phosphate and also 2-deoxy-d-ribose-5-phosphate, 2-deoxy-d-glucose-6-phosphate, d-mannose-6-phosphate, d-fructose-1-phosphate, d-glucose-6-phosphate, dl-glycerol-3-phosphate, and d-fructose-6-phosphate. The kinetic parameters show a humble specificity and efficiency that resemble those of the bacterial BT4131, which is a member of the HAD subfamily IIB with a C2B-type cap domain. BT4131 biochemical function was identified by the conjoined information from residues stationed on substrate specificity loops, the active site solvent cage and the genome context of the encoding gene (Lu et al. 2005) while for AtSgpp, instead of the genomic context, was the close homology with *Arabidopsis* dl-glycerol-3-phosphatase AtGpp who designed the sugar phosphoesters screening. AtSgpp phosphatase activity is optimal at pH 7.0, albeit fairly pH independent on d-ribose-5-phosphate cleaving. At present, it is not known if the ribose ester is the main natural hydrolyzing substrate of AtSgpp, or if it could be another non-screened candidate. Also, if using different assay conditions, the *Arabidopsis* homologous At4g39970, At3g48420 and At2g33255 will cleave sugar phosphomonoesters, or if they hydrolyze different substrates. *AtSgpp* is ubiquitously expressed throughout development and its expression is affected by abiotic and biotic stresses, being the greatest under Pi starvation and cyclopentenone oxylipins induction. Therefore, taking into account both the activity and the expression data, the AtSgpp physiological function appears to be somehow related to housekeeping detoxification, sugar-phosphate modulation and in maintaining the homeostatic balance of Pi in the cell. Another aim of this work was to provide further information as to the relationship that binds the function to the substrate specificity loop, to contribute to answer the formerly enunciated question about how then do HAD phosphohydrolases distinguish their substrates? It could be claimed that functional peers can be thereby identified by their cap domain signature-sequence motif, though this function assignment would remain constrained to a single kingdom.

## Abbreviations

HAD: Haloacid dehalogenase-like hydrolase proteins
MBP: Maltose-binding protein

## References

[CR1] Allen KN, Dunaway-Mariano D (2004). Phosphoryl group transfer: evolution of a catalytic scaffold. Trends Biochem Sci.

[CR2] Altschul SF, Gish W, Miller W, Myers EW, Lipman DJ (1990). Basic local alignment search tool. J Mol Biol.

[CR3] Ames BN (1966). Assay of inorganic phosphate, total phosphate, and phosphatases. Methods Enzymol.

[CR4] Böhmer M, Schroeder JI (2011). Quantitative transcriptomic analysis of abscisic acid-induced and reactive oxygen species-dependent expression changes and proteomic profiling in Arabidopsis suspension cells. Plant J.

[CR5] Bradford MM (1976). A rapid and sensitive method for the quantization of microgram quantities of protein utilizing the principle of protein-dye binding. Anal Biochem.

[CR6] Burroughs AM, Allen KN, Dunaway-Mariano D, Aravind L (2006). Evolutionary genomics of the HAD superfamily: understanding the structural adaptations and catalytic diversity in a superfamily of phosphoesterases and allied enzymes. J Mol Biol.

[CR7] Caparrós-Martín JA, Reiland S, Köchert K, Cutanda MC, Culiáñez-Macia FA (2007). *Arabidopsis thaliana* AtGpp 1 and AtGpp2: two novel low molecular weight phosphatases involved in plant glycerol metabolism. Plant Mol Biol.

[CR8] Collet JF, Stroobant V, Pirard M, Delpierre G, Van Schaftingen E (1998). A new class of phosphotransferases phosphorylated on an aspartate residue in an amino-terminal DXDX(T/V) motif. J Biol Chem.

[CR9] Corpet F, Servantm F, Gouzy J, Kahn D (2000). ProDom and ProDom-CG: tools for protein domain analysis and whole genome comparisons. Nucleic Acids Res.

[CR10] Cutanda MC (2003) Effect of altering levels of hexoses phosphate in carbohydrate metabolism and glucose signalling in yeast and plants. PhD thesis, Polytechnic University of Valencia, Valencia, Spain

[CR11] Higgins D, Thompson J, Gibson T, Thompson JD, Higgins DG, Gibson TJ (1994). CLUSTAL W: improving the sensitivity of progressive multiple sequence alignment through sequence weighting, position-specific gap penalties and weight matrix choice. Nucleic Acids Res.

[CR12] Koonin EV, Tatusov RL (1994). Computer analysis of bacterial haloacid dehalogenases defines a large superfamily of hydrolases with diverse specificity. Application of an iterative approach to database search. J Mol Biol.

[CR13] Kupke T, Caparrós-Martín JA, Malquichagua Salazar KJ, Culiàñez-Macià FA (2009). Biochemical and physiological characterization of *Arabidopsis thaliana* AtCoAse: a Nudix CoA hydrolyzing protein that improves plant development. Physiol Plant.

[CR14] Kuznetsova E, Proudfoot M, Sanders SA, Reinking J, Savchenko A, Arrowsmith CH, Edwards AM, Yakunin AF (2005). Enzyme genomics: application of general enzymatic screens to discover new enzymes. FEMS Microbiol Rev.

[CR15] Kuznetsova E, Proudfoo M, Gonzalez CF, Brown G, Omelchenko MV, Borozan I, Carmel L, Wolf YI, Mori H, Savchenko AV, Arrowsmith CH, Koonin EV, Edwards AM, Yakunin AF (2006). Genome-wide analysis of substrate specificities of the *Escherichia coli* haloacid dehalogenase-like phosphatase family. J Biol Chem.

[CR16] Lahiri SD, Zhang G, Dai J, Dunaway-Mariano D, Allen KN (2004). Analysis of the substrate specificity loop of the HAD superfamily cap domain. Biochemistry.

[CR17] Lahiri SD, Zhang G, Dunaway-Mariano D, Allen KN (2006). Diversification of function in the haloacid dehalogenase enzyme superfamily: the role of the cap domain in hydrolytic phosphorus—carbon bond cleavage. Bioorganic Chem.

[CR18] Lambert C, Leonard N, De Bolle X, Depiereux E (2002). ESyPred3D: prediction of proteins 3D structures. Bioinformatics.

[CR19] Lu Z, Dunaway-Mariano D, Allen KN (2005). HAD superfamily phosphotransferase substrate diversification: structure and function analysis of HAD subclass IIB sugar phosphatase BT4131. Biochemistry.

[CR20] Lu Z, Dunaway-Mariano D, Allen KN (2008). The catalytic scaffold of the haloalkanoic acid dehalogenase enzyme superfamily acts as a mold for the trigonal bipyramidal transition state. Proc Natl Acad Sci USA.

[CR21] Maniatis T, Fritsch EF, Sambrook J (1982). Molecular cloning: a laboratory manual.

[CR22] Morais MC, Zhang W, Baker AS, Zhang G, Dunaway-Mariano D, Allen KN (2000). The crystal structure of *Bacillus cereus* phosphonoacetaldehyde hydrolase: insight into catalysis of phosphorus bond cleavage and catalytic diversification within the HAD enzyme superfamily. Biochemistry.

[CR23] Morais MC, Zhang G, Zhang W, Olsen DB, Dunaway-Mariano D, Allen KN (2004). X-ray crystallographic and site-directed mutagenesis analysis of the mechanism of Schiff-base formation in phosphonoacetaldehyde hydrolase catalysis. J Biol Chem.

[CR24] Mueller WS, Hilbert B, Dueckershoff K, Roitsch T, Krischke M, Mueller MJ, Berger S (2008). General detoxification and stress responses are mediated by oxidized lipids through TGA transcription factors in *Arabidopsis*. Plant Cell.

[CR25] Murashige T, Skoog F (1962). A revised medium for rapid growth and bioassays with tobacco cultures. Physiol Plant.

[CR26] Norbeck J, Pahlman AK, Akhtar N, Blomberg A, Adler L (1996). Purification and characterization of two isoenzymes of dl-glycerol-3-phosphatase from *Saccharomyces cerevisiae*. Identification of the corresponding GPP1 and GPP2 genes and evidence for osmotic regulation of Gpp 2p expression by the osmosensing mitogen-activated protein kinase signal transduction pathway. J Biol Chem.

[CR27] Rández-Gil F, Blasco A, Prieto JA, Sanz P (1995). *DOGR1* and *DOGR2*: two genes from *Saccharomyces cerevisiae* that confer 2-deoxyglucose resistance when overexpressed. Yeast.

[CR28] Rao KN, Kumaran D, Seetharaman J, Bonanno JB, Burley SK, Swaminathan S (2006). Crystal structure of trehalose-6-phosphate phosphatase-related protein: biochemical and biological implications. Protein Sci.

[CR29] Schagger H, von Jagow G (1987). Tricine-sodium dodecyl sulfatepolyacrylamide gel electrophoresis for the separation of proteins in the range from 1 to 100 kDa. Anal Biochem.

[CR30] Schenk PM, Baumann S, Mattes R, Steinbiss HH (1995). Improved high-level expression system for eukaryotic genes in *Escherichia coli* using T7 RNA polymerase and rare ArgtRNAs. Biotechniques.

[CR31] Selengut JD (2001). MDP-1 is a new and distinct member of the haloacid dehalogenase family of aspartate-dependent phosphohydrolases. Biochemistry.

[CR32] Selengut JD, Levine RL (2000). MDP-1: a novel eukaryotic magnesium-dependent phosphatase. Biochemistry.

[CR33] Shin DH, Roberts A, Jancarik J, Yocota H, Kim R, Wemmer DE, Kim S-H (2003). Crystal structure of a phosphatase with a unique substrate binding domain from *Thermotoga maritime*. Protein Sci.

[CR34] Sussman I, Avron M (1981). Characterization and partial puri-fication of dl-glycerol-1-phosphatase from *Dunaliella salina*. Biochim Biophys Acta.

[CR35] The Arabidopsis Genome Initiative (2000). Analysis of the genome sequence of the flowering plant *Arabidopsis thaliana*. Nature.

[CR36] Tremblay LW, Dunaway-Mariano D, Allen KN (2006). Structure and activity analyses of *Escherichia coli* K-12 NagD provide insight into the evolution of biochemical function in the haloalkanoic acid dehalogenase superfamily. Biochemistry.

[CR37] Vicient CM, Delseny M (1999). Isolation of total RNA from *Arabidopsis thaliana* seeds. Anal Biochem.

[CR38] Wang W, Cho HS, Kim R, Jancarik J, Yokota H, Nguyen HH, Grigoriev IV, Wemmer DE, Kim S-H (2002). Structural characterization of the reaction pathway in phosphoserine phosphatase: crystallographic “snapshots” of intermediate states. J Mol Biol.

[CR39] Zhang G, Mazurkie AS, Dunaway-Mariano D, Allen KN (2002). Kinetic evidence for a substrate-induced fit in phosphonoacetaldehyde hydrolase catalysis. Biochemistry.

[CR40] Zhang G, Morais MC, Dai J, Zhang W, Dunaway-Mariano D, Allen KN (2004). Investigation of metal Ion binding in phosphonoacetaldehyde hydrolase identifies sequence markers for metal-activated enzymes of the HAD enzyme superfamily. Biochemistry.

[CR41] Zimmermann P, Hirsch-Hoffmann M, Hennig L, Gruissem W (2004). GENEVESTIGATOR: Arabidopsis microarray database and analysis toolbox. Plant Physiol.

